# Supplementary figures and images for: Phosphorylation of mouse intestinal basolateral amino acid uniporter LAT4 is controlled by food-entrained diurnal rhythm and dietary proteins
Source: PLoS One. 2020 May 29;15(5):e0233863. doi: 10.1371/journal.pone.0233863 (PMC7259769; doi:10.1371/journal.pone.0233863)

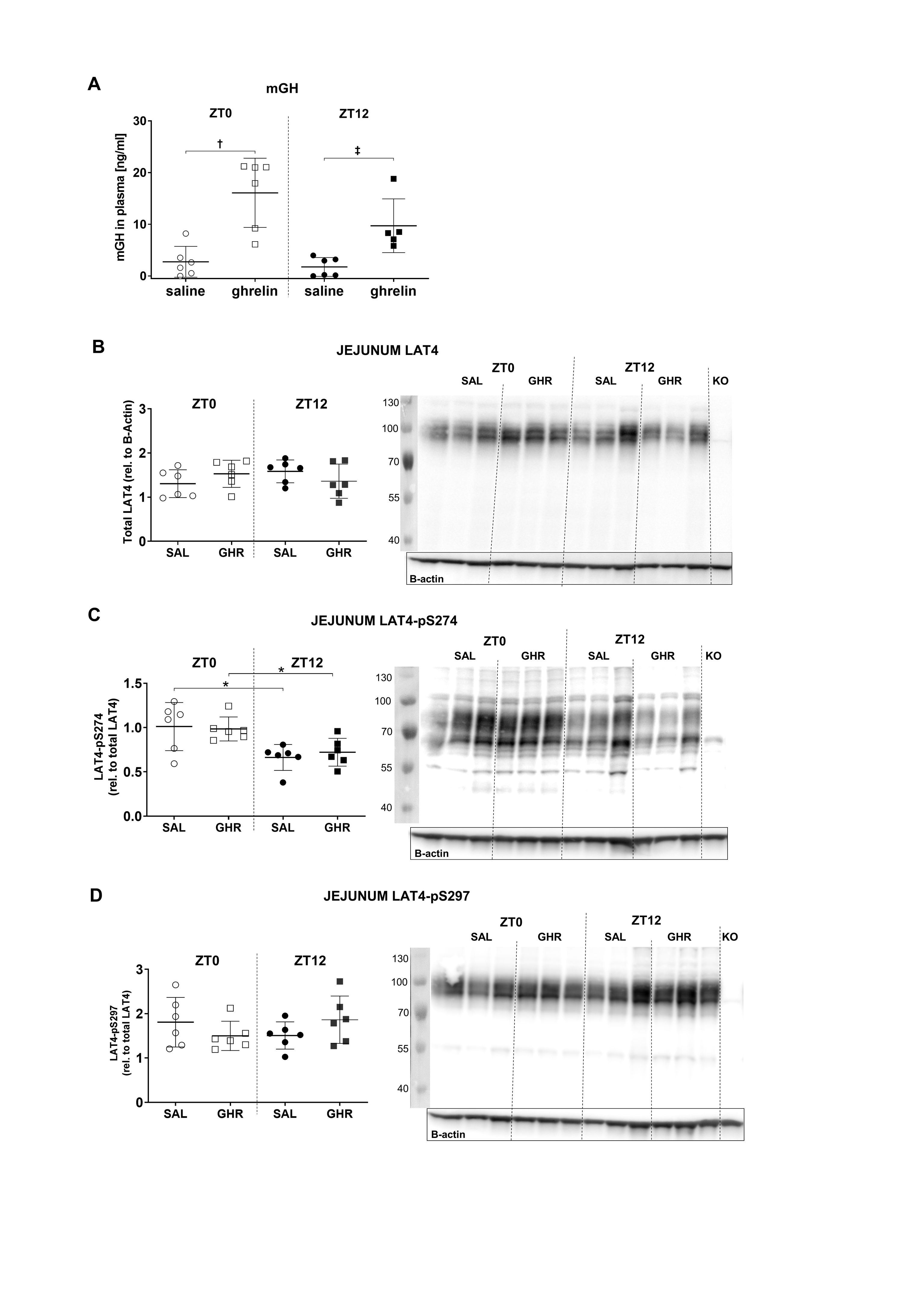

Supplement: S1 Fig — Food-entrained mice received an injection with either pure saline or saline with 10 μg ghrelin and were sacrificed 10 min later. A: Concentration of growth hormone in mouse plasma determined the efficiency of ghrelin injections. B: LAT4 protein expression in jejunum. C: LAT4 phosphorylation on S274 in jejunum. D: LAT4 phosphorylation on S297 in jejunum. B to D: Total lysates from intestinal villi fraction of WT and LAT4 conditional KO mice analyzed by Western blot. For quantification, all values were normalized to beta actin. Further normalization to LAT4 ratio was done for phospho-specific antibodies. Quantification is shown in the left panel, representative blot in the right panel. Statistical analysis performed with One-way ANOVA using Tukey’s multiple comparison test, *p<0.05. Mean (SD), n = 6 mice per injection group from single experiment. (TIF) [file pone.0233863.s001.tif]
