# Supplementary material for: Phosphorylation of mouse intestinal basolateral amino acid uniporter LAT4 is controlled by food-entrained diurnal rhythm and dietary proteins
Source: PLoS One. 2020 May 29;15(5):e0233863. doi: 10.1371/journal.pone.0233863 (PMC7259769; doi:10.1371/journal.pone.0233863)
Supplement: S1 Raw Images — Compilation of all original blot images. Panels that were used for corresponding figures as well as the additional modifications are indicated. (PDF) [file pone.0233863.s002.pdf]

Original blot images from Fig 2A

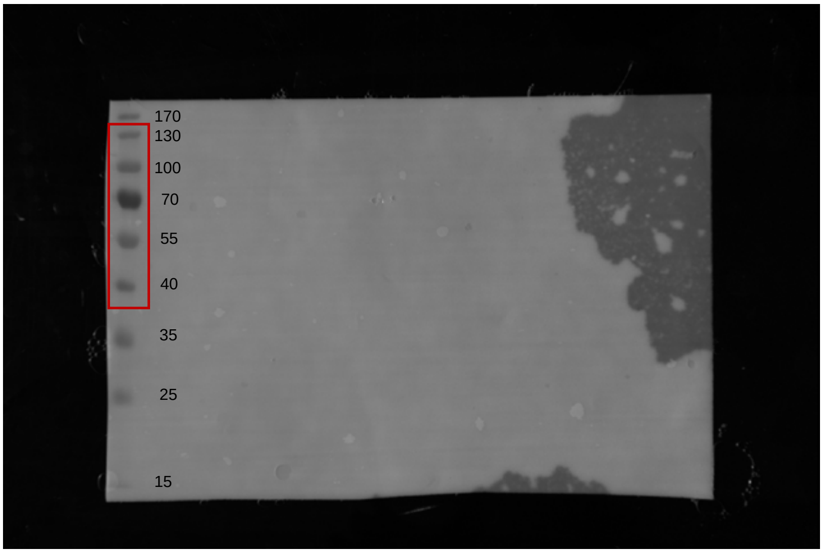

Marker, showing protein weight in kDa. Adjustment used in Fig 2A: increased contrast (+40%) and brightness (+20%), cropped between 130 and 40 kDa, vertical extension.

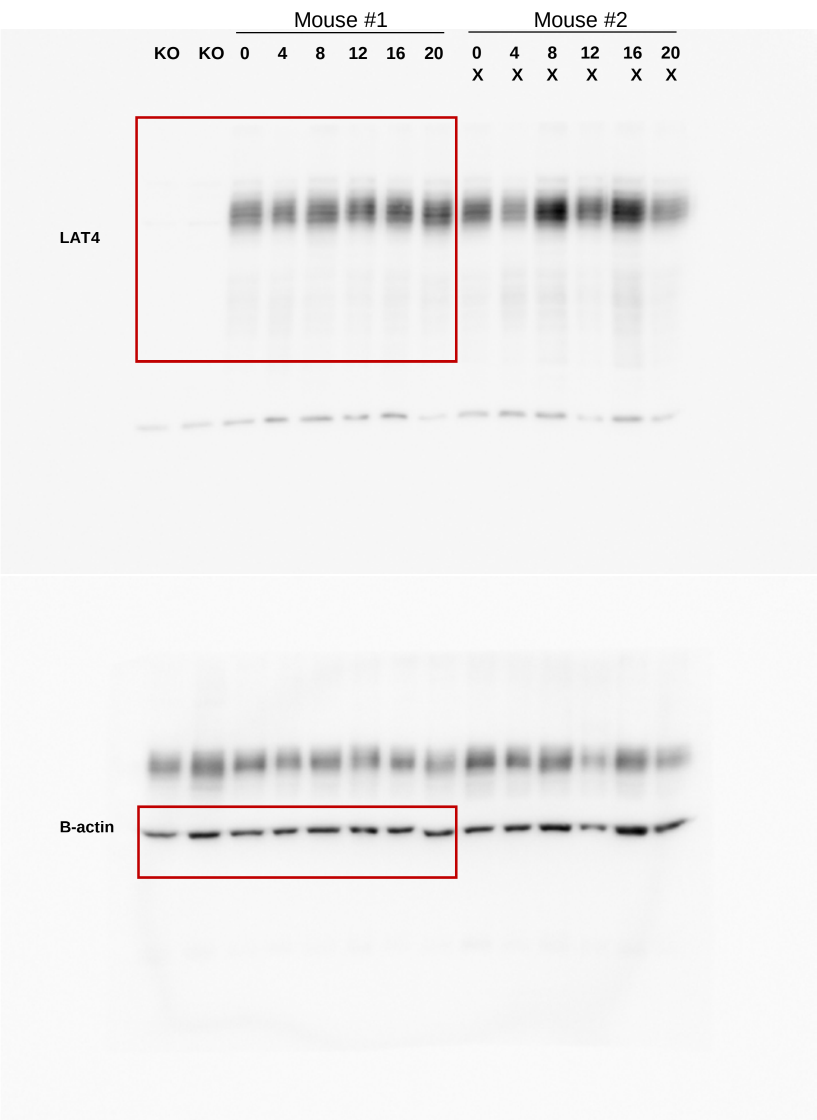

LAT4 and actin original blot images. Loading sequence shown, numbers show ZT timepoint. X marks the lanes not included in the Fig 2A. Adjustments used in Fig 2A: contrast increased by 20%, cropped between 130 and 40 kDa, vertical extension for LAT4.

Original blot images from Fig 2B

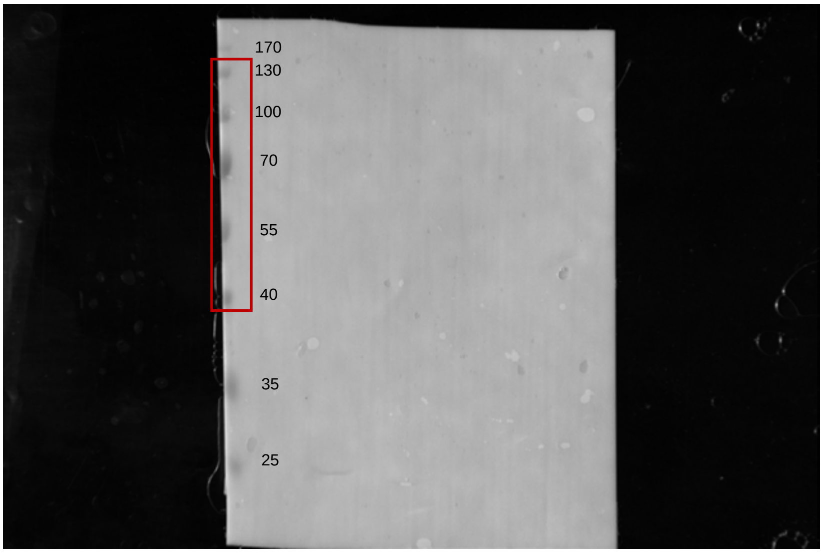

Marker, showing protein weight in kDa. Adjustments used in Fig 2B: increased contrast (+40%) and brightness (+20%), cropped between 130 and 40 kDa, vertical extension.

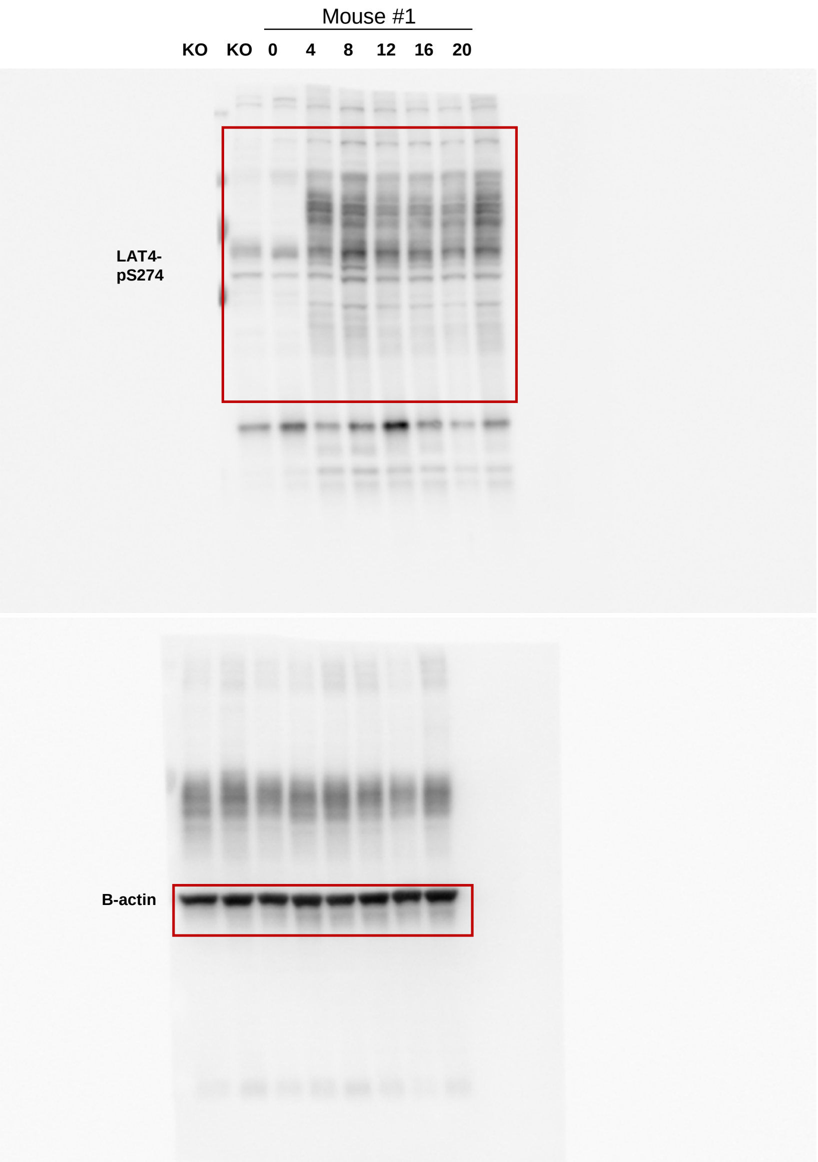

LAT4-pS274 and actin original blot images. Loading sequence shown, numbers show ZT timepoint. Adjustments used in Fig 2B: cropped between 130 and 40 kDa, vertical extension.

Original blot images from Fig 2C

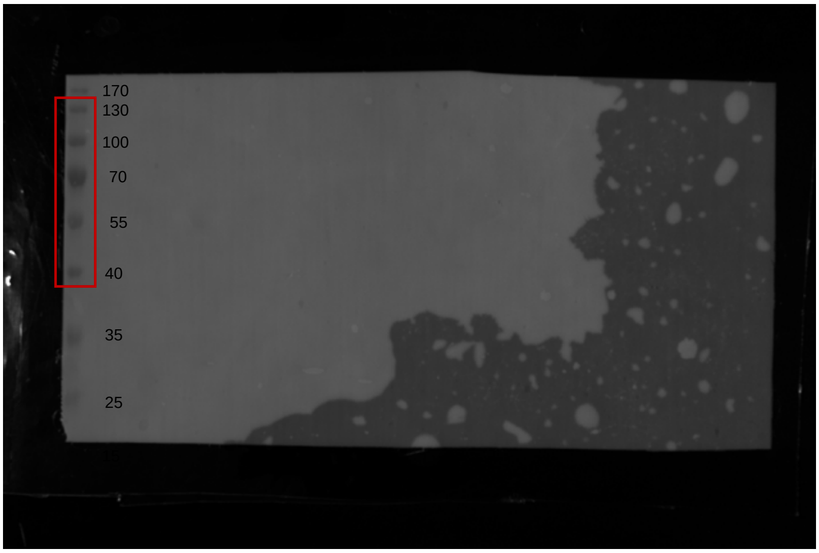

Marker, showing protein weight in kDa. Adjustment used in Fig 2C: increased contrast (+40%) and brightness (+20%), cropped between 130 and 40 kDa, vertical extension.

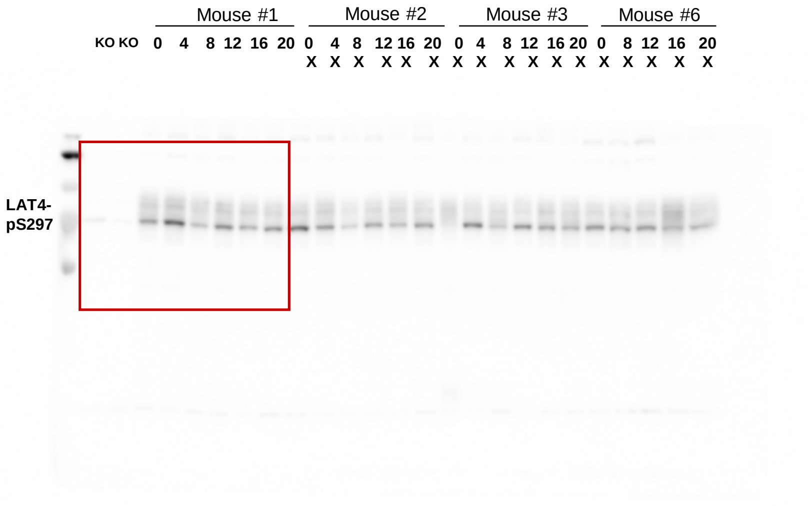

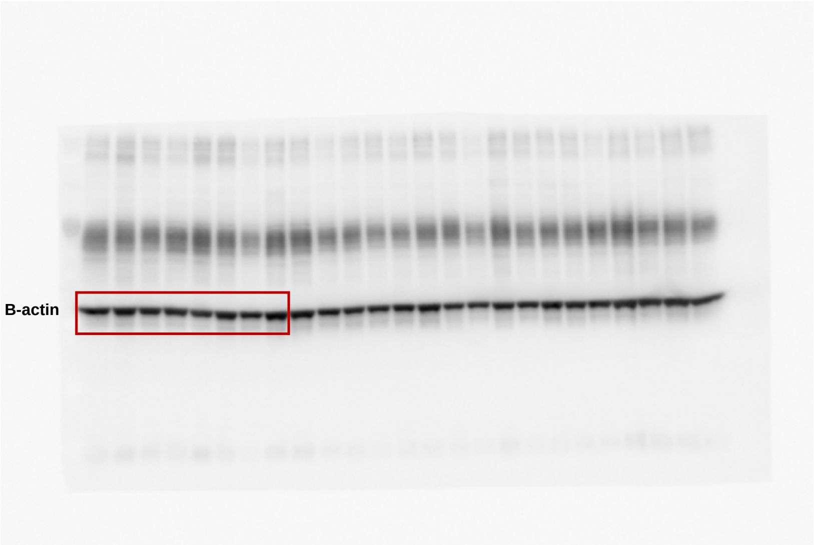

LAT4-pS297 and actin original blot images. Loading sequence shown, numbers show ZT timepoint. X marks the lanes not included in the Fig 2C. Adjustments used in Fig 2C: contrast increased by 20%, images darkened, cropped between 130 and 40 kDa, vertical extension for LAT4-pS297.

Original blot images from Fig 3A

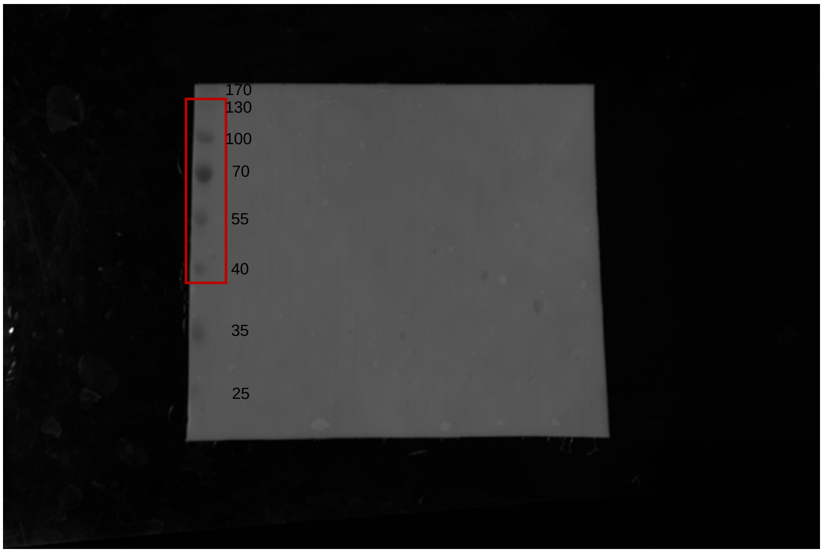

Marker, showing protein weight in kDa. Adjustment used in Fig 3A: increased contrast (+40%) and brightness (+40%), cropped between 130 and 40 kDa, vertical extension.

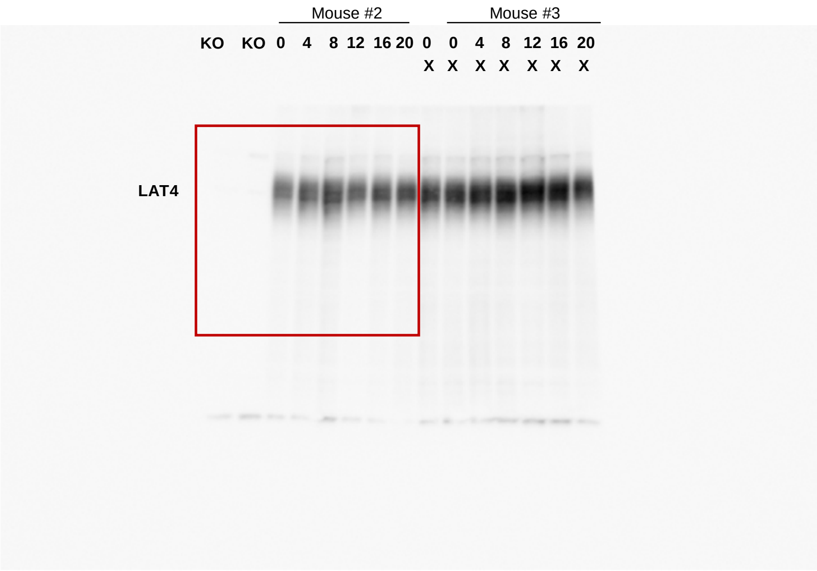

Sample “0” between mouse #2 and mouse #3 is a sample from mouse #1 taken at ZT0, that was loaded on all the blots from timepoint experimental set as additional control sample to assess individual variation.

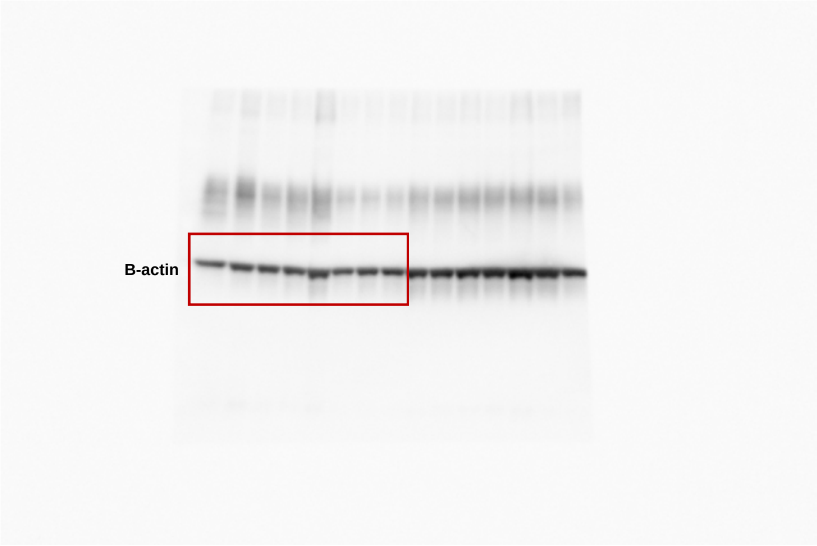

LAT4 and actin original blot images. Loading sequence shown, numbers show ZT timepoint. X marks the lanes not included in the Fig 3A. Adjustments used in Fig 3A: cropped between 130 and 40 kDa, vertical extension for LAT4.

Original blot images from Fig 3B

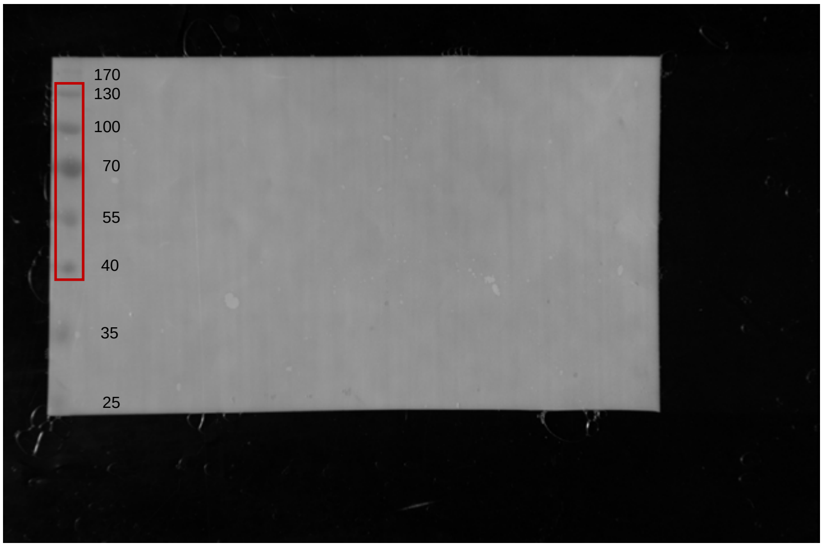

Marker, showing protein weight in kDa. Adjustment used in Fig 3B: increased contrast (+40%) and brightness (+20%), cropped between 130 and 40 kDa, vertical extension.

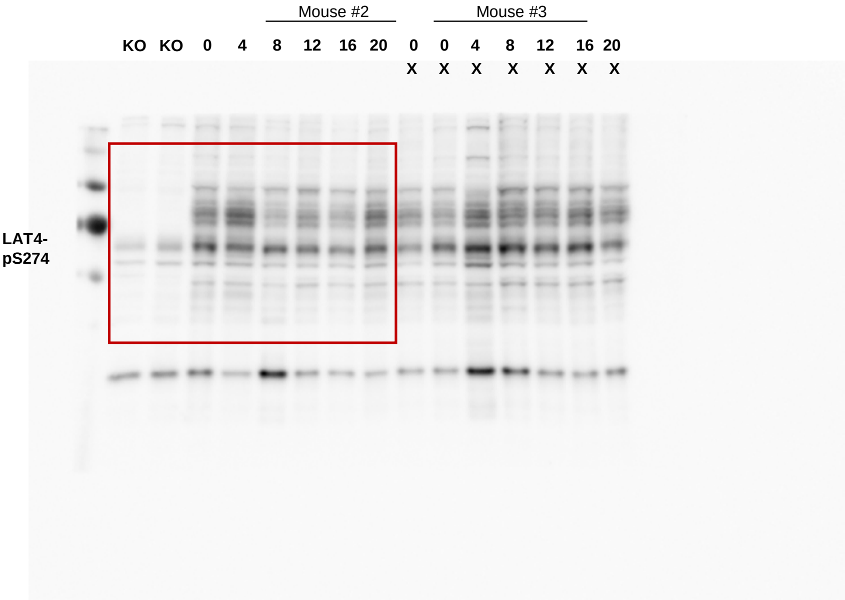

Sample "0" between mouse #2 and mouse #3 is a sample from mouse #1 taken at ZT0, that was loaded on all the blots from timepoint experimental set as additional control sample to assess individual variation.

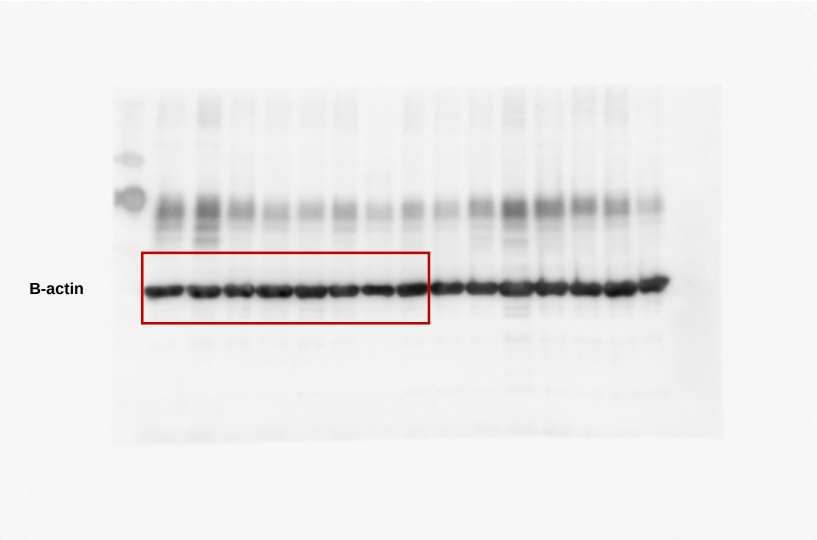

LAT4-pS274 and actin original blot images. Loading sequence shown, numbers show ZT timepoint. X marks the lanes not included in the Fig 3B. Adjustments used in Fig 3B: cropped between 130 and 40 kDa, vertical extension for LAT4.

Original blot images from Fig 3C

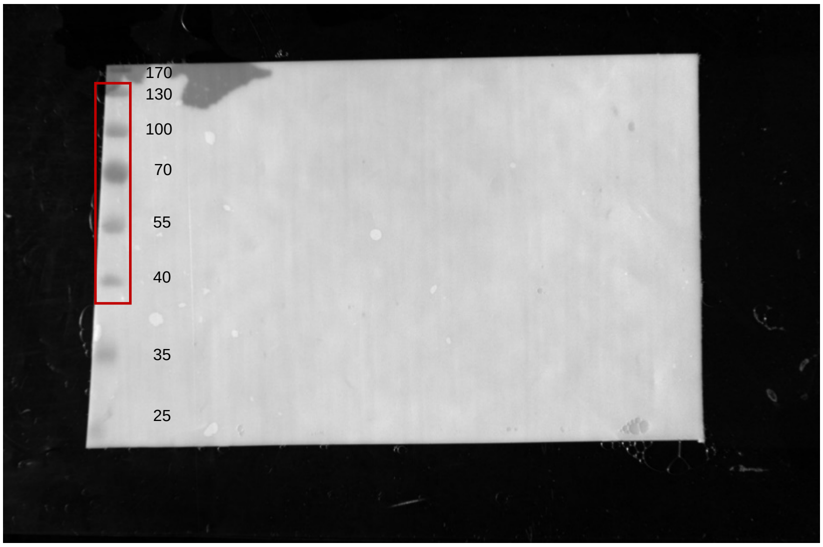

Marker, showing protein weight in kDa. Adjustment used in Fig 3C: increased contrast (+20%), cropped between 130 and 40 kDa, vertical extension.

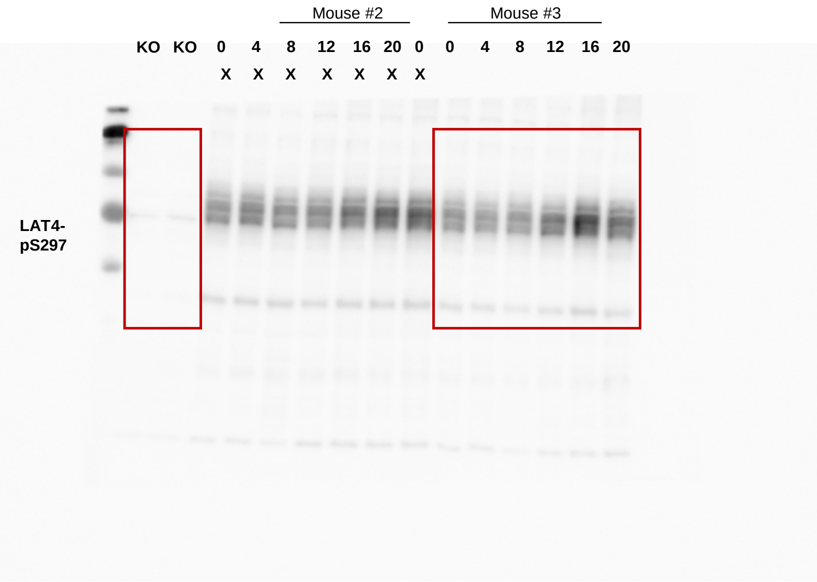

Sample “0” between mouse #2 and mouse #3 is a sample from mouse #1 taken at ZT0, that was loaded on all the blots from timepoint experimental set as additional control sample to assess individual variation

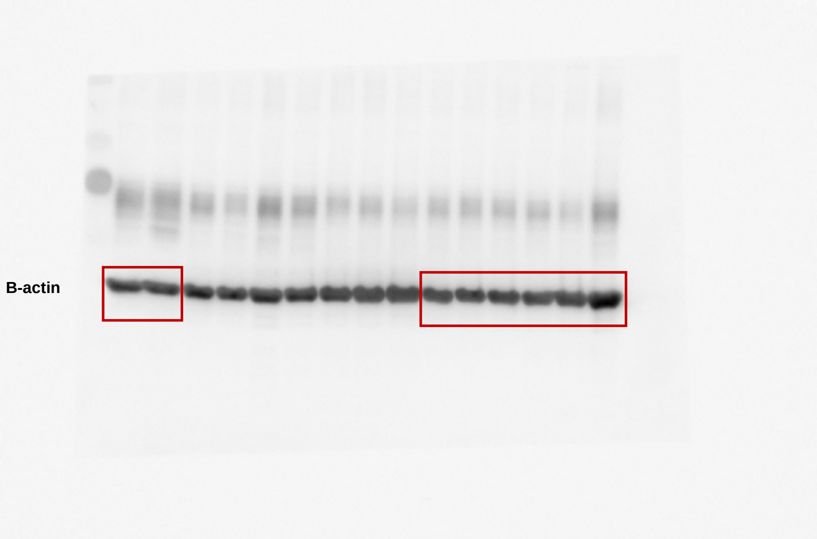

LAT4-pS297 and actin original blot images. Loading sequence shown, numbers show ZT timepoint. X marks the lanes not included in the Fig 3C. Adjustments used in Fig 3C: cropped between 130 and 40 kDa, vertical extension for LAT4.

Original blot images from Fig 12A

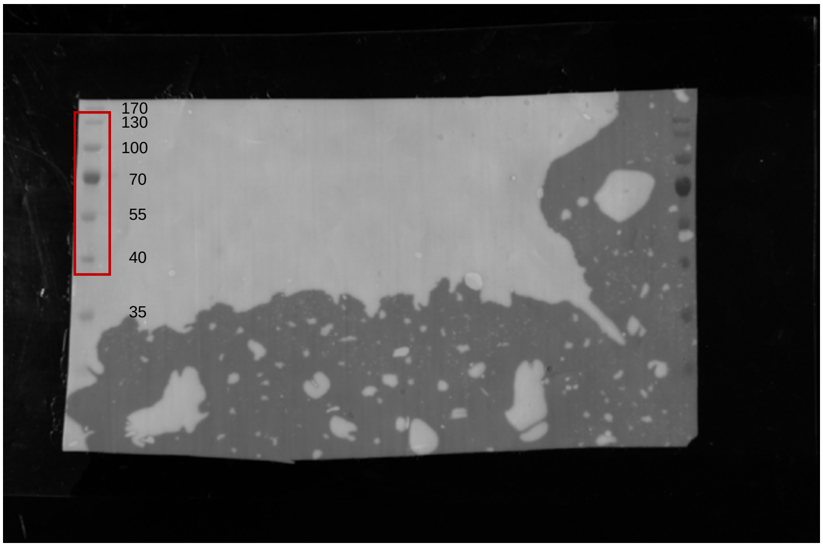

Marker, showing protein weight in kDa. Adjustment used in Fig 12A: increased contrast (+20%) and brightness (+20%), cropped between 130 and 40 kDa, vertical extension.

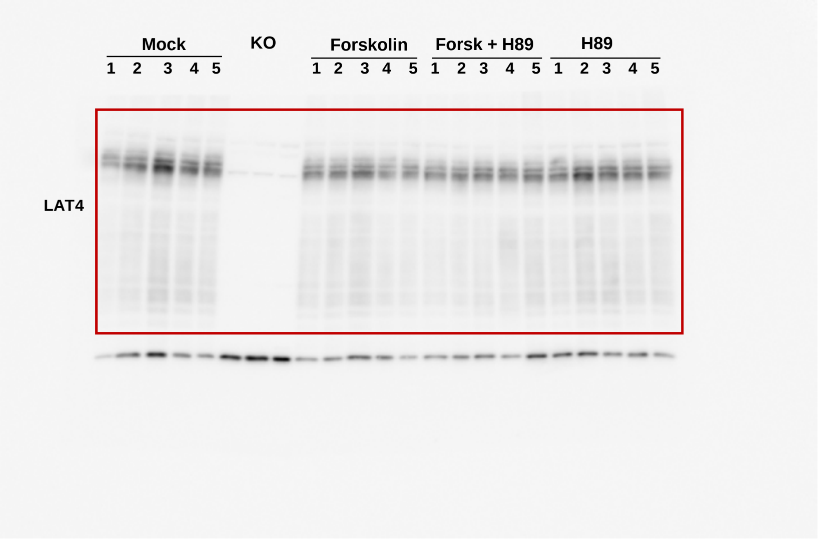

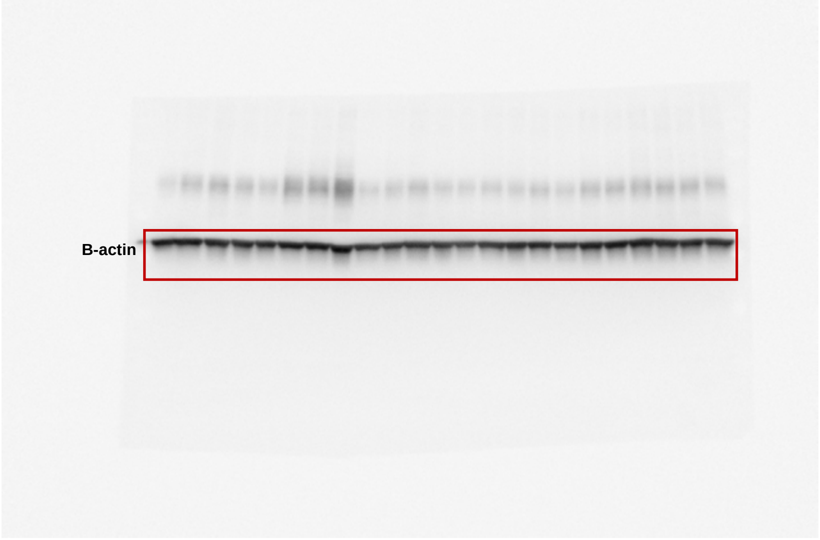

LAT4 and actin original blot images. Loading sequence shown, same sample numbers indicate same mouse tissue. Adjustments used in Fig 12A: cropped between 130 and 40 kDa, vertical compression and increased contrast (20%) and darkened for LAT4.

Original blot images from Fig 12B

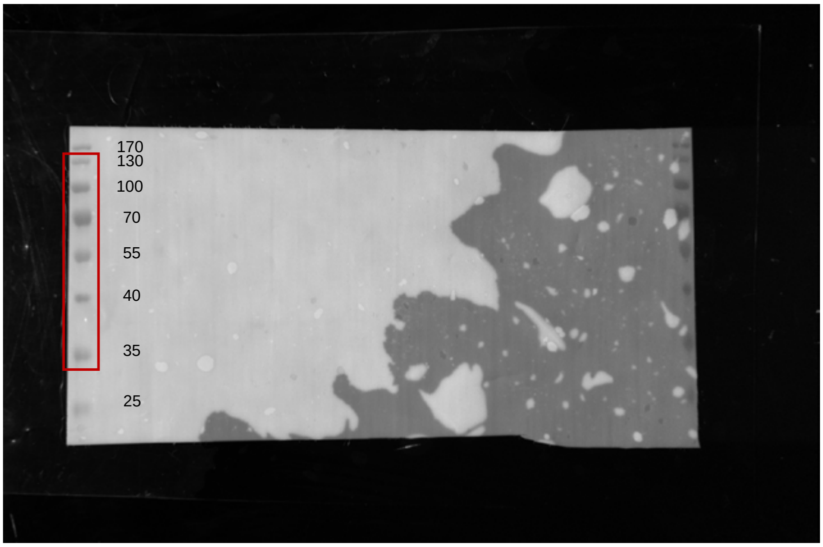

Marker, showing protein weight in kDa. Adjustment used in Fig 12B: increased contrast (+40%), cropped between 130 and 35 kDa, vertical extension.

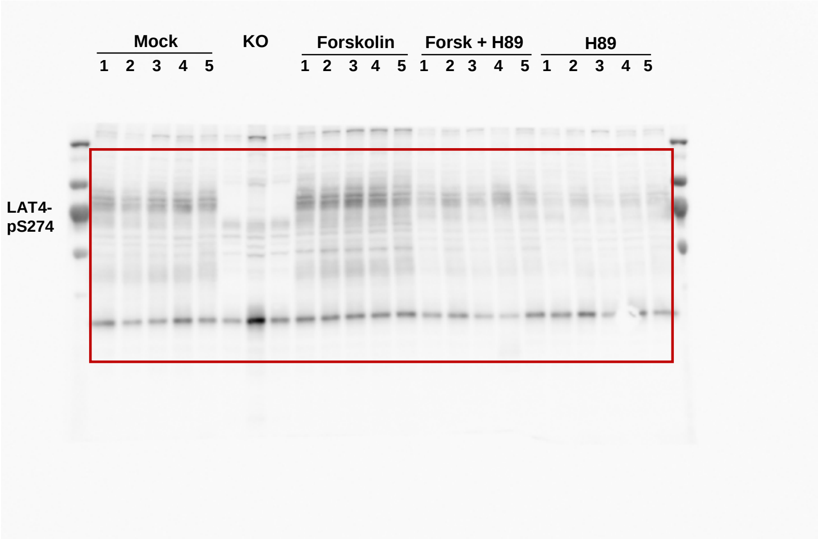

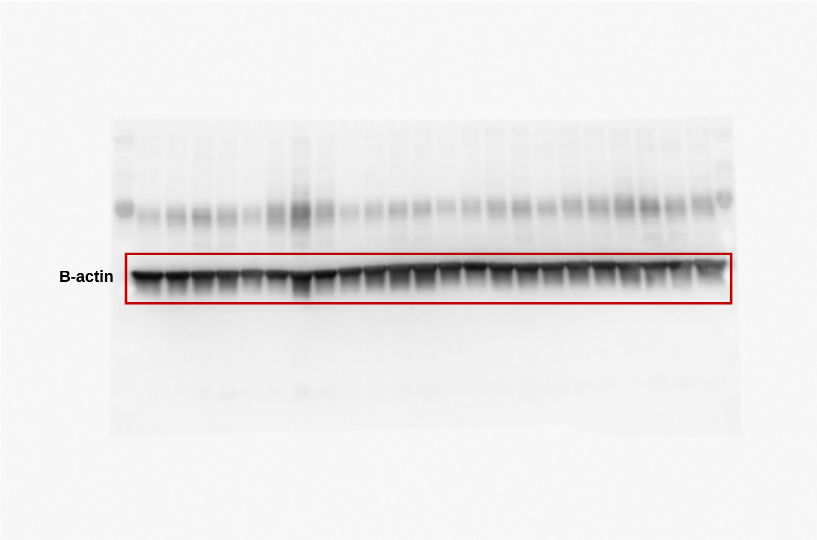

LAT4-pS274 and actin original blot images. Loading sequence shown, same sample numbers indicate same mouse tissue. Adjustments used in Fig 12B: cropped between 130 and 35 kDa, vertical compression and increased contrast (20%) and darkened for LAT4-pS274. Vertical compression for actin.

Original blot images from Fig 12C

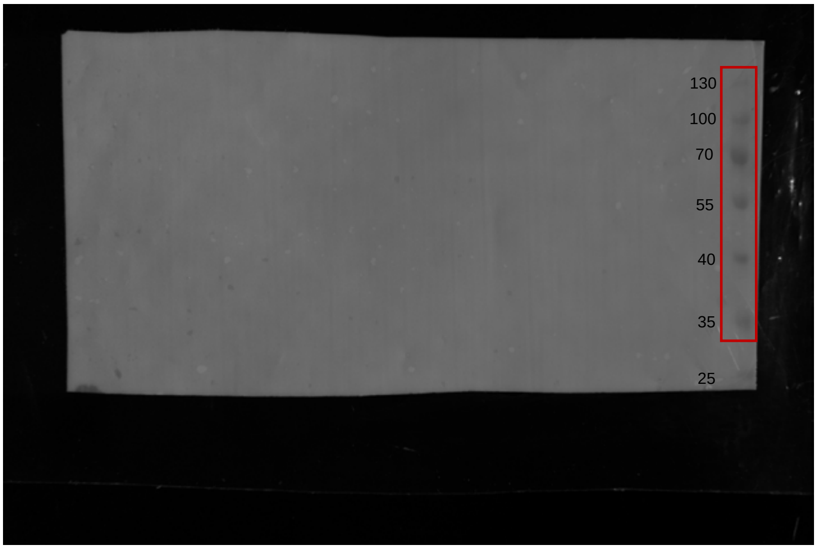

Marker, showing protein weight in kDa. Adjustment used in Fig 12C: increased contrast (+40%) and brightness (+20%), cropped between 130 and 35 kDa, vertical extension.

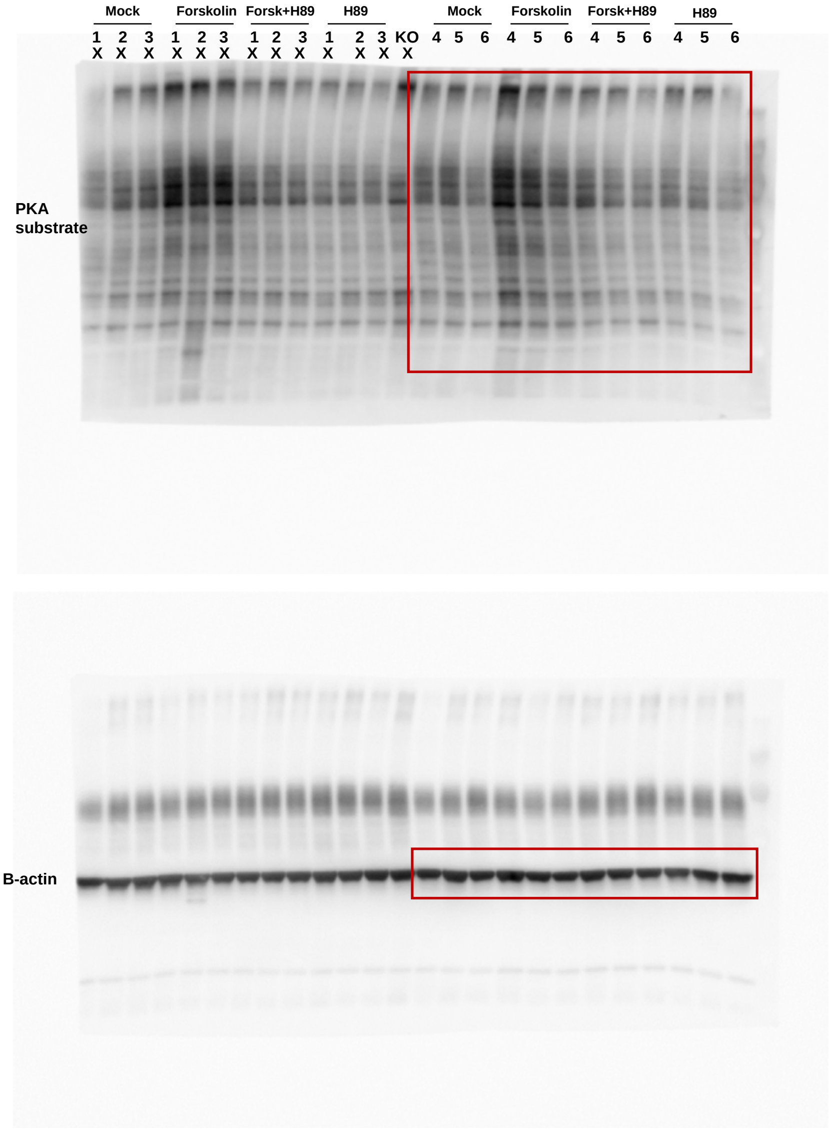

PKA substrate and actin original blot images. Loading sequence shown, same sample numbers indicate same mouse tissue. X marks the lanes not included in the Fig 12C. Adjustments used in Fig 12C: cropped between 130 and 35 kDa, vertical compression for both and increased contrast (20%) for PKA substrate.

Original blot images from Fig 12D

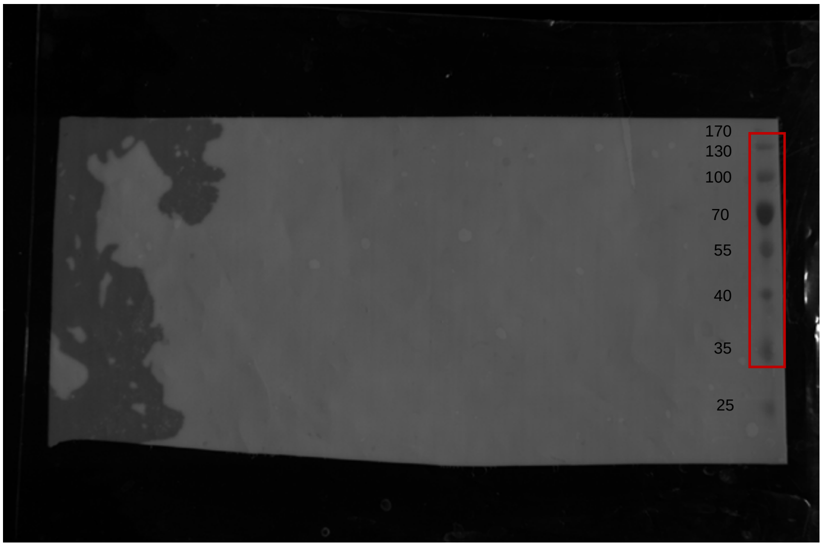

Marker, showing protein weight in kDa. Adjustment used in Fig 12D: increased contrast (+40%) and brightness (+40%), cropped between 130 and 35 kDa, vertical extension.

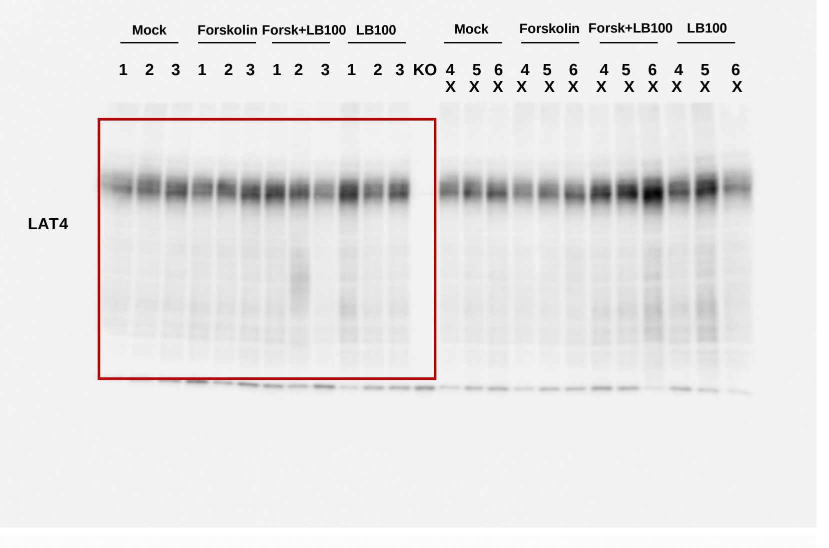

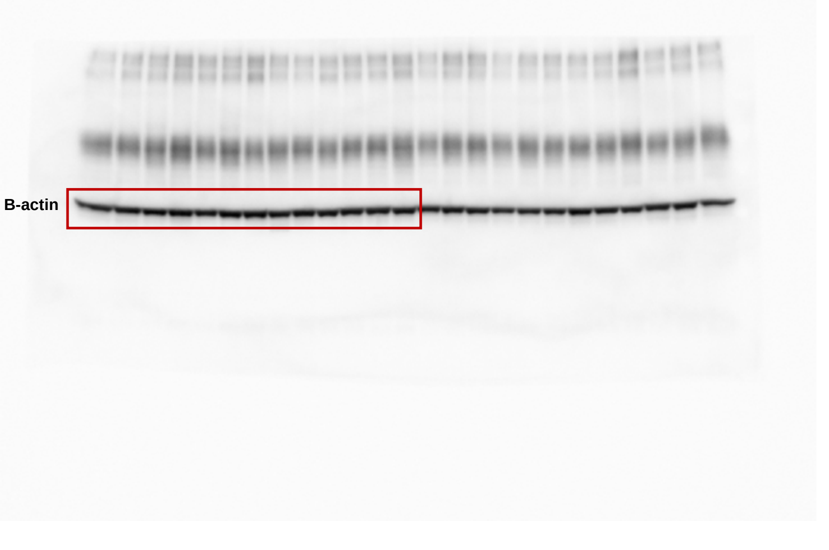

LAT4 and actin original blot images. Loading sequence shown, same sample numbers indicate same mouse tissue. X marks the lanes not included in the Fig 12D. Adjustments used in Fig 12D: cropped between 130 and 35 kDa, vertical compression and increased contrast (20%) for LAT4.

Original blot images from Fig 12E

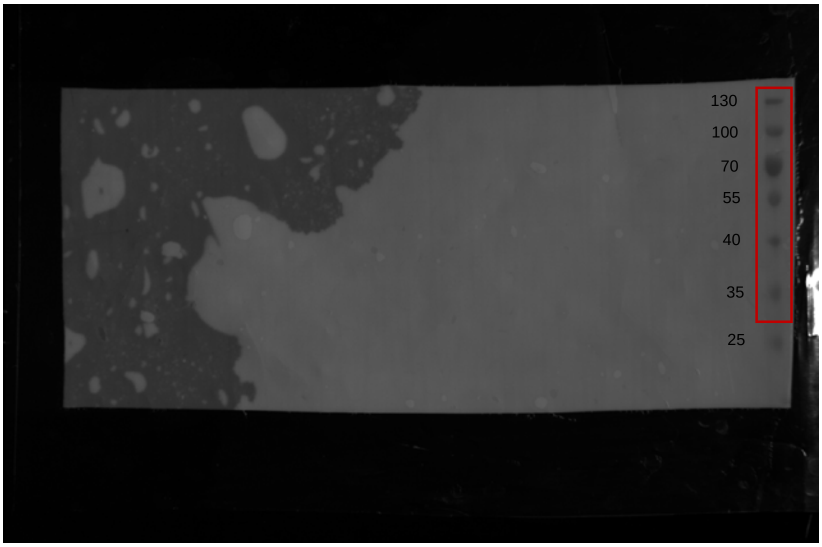

Marker, showing protein weight in kDa. Adjustment used in Fig 12E: increased contrast (+40%) and brightness (+40%), cropped between 130 and 35 kDa, vertical extension.

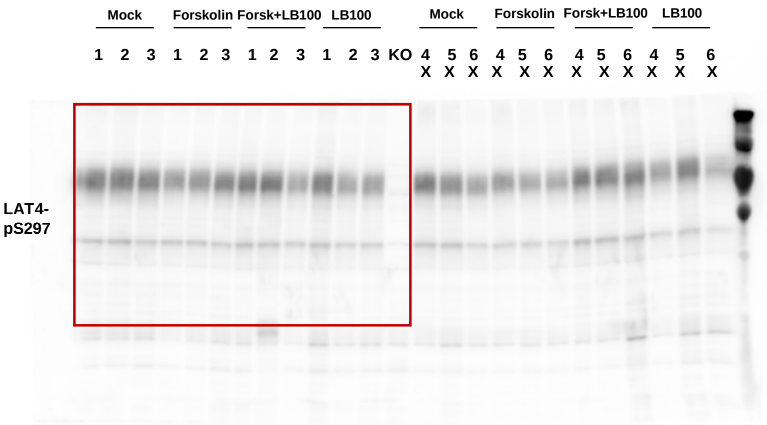

LAT4-pS297 and actin original blot images. Loading sequence shown, same sample numbers indicate same mouse tissue. X marks the lanes not included in the Fig 12E. Adjustments used in Fig 12E: cropped between 130 and 35 kDa, vertical extension and increased contrast (20%) for LAT4-pS297. Vertical compression for actin.

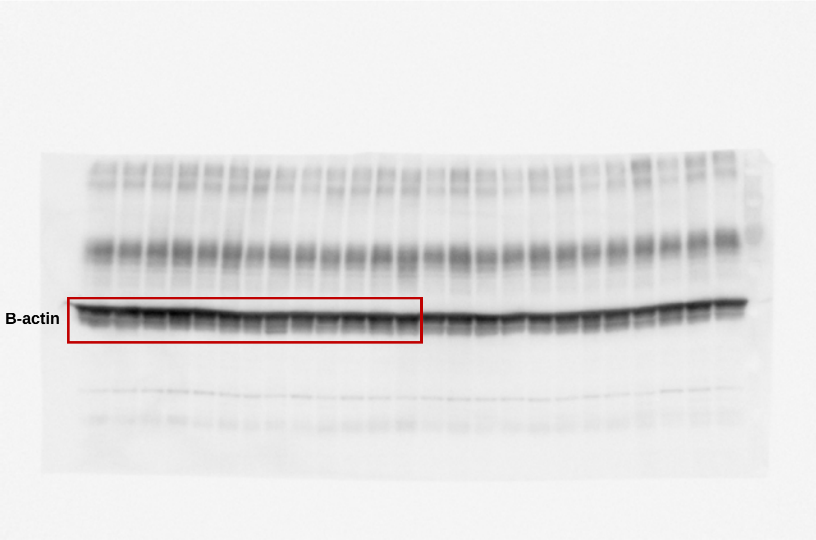

Original blot images from Fig 13A

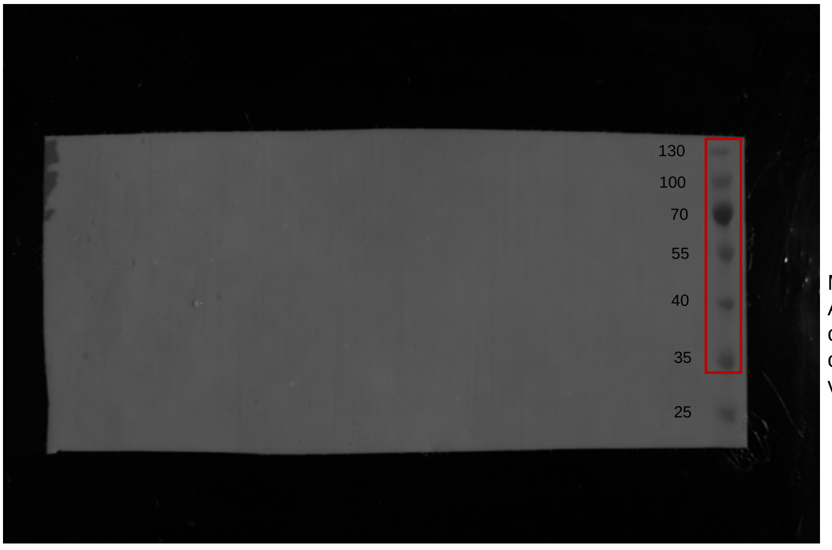

Marker, showing protein weight in kDa. Adjustment used in Fig 13A: increased contrast (+20%) and brightness (+40%), cropped between 130 and 35 kDa, vertical extension.

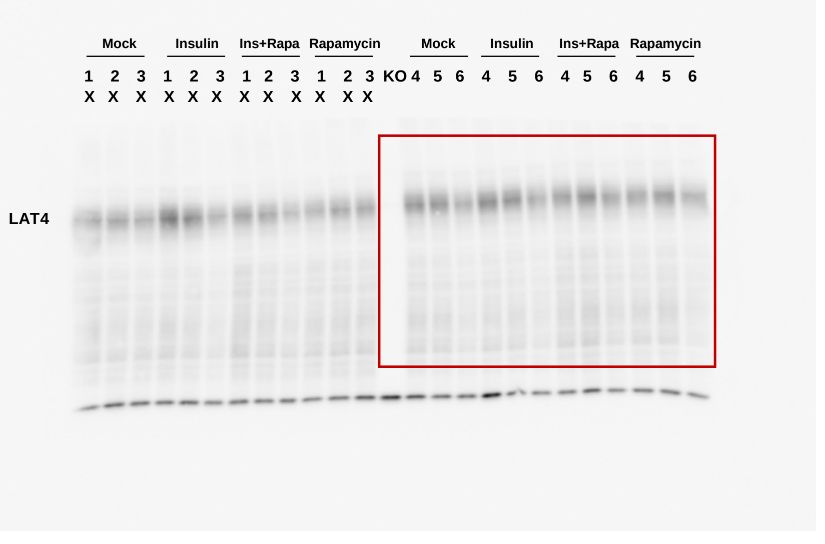

LAT4 and actin original blot images. Loading sequence shown, same sample numbers indicate same mouse tissue. X marks the lanes not included in the Fig 13A. Adjustments used in Fig 13A: cropped between 130 and 35 kDa, vertical compression and increased contrast (20%) and darkening for LAT4. Vertical compression for actin.

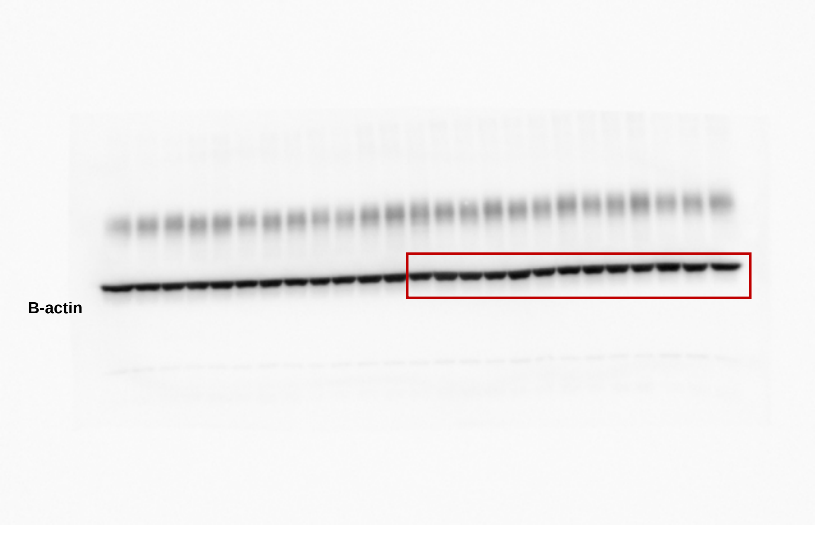

Original blot images from Fig 13B

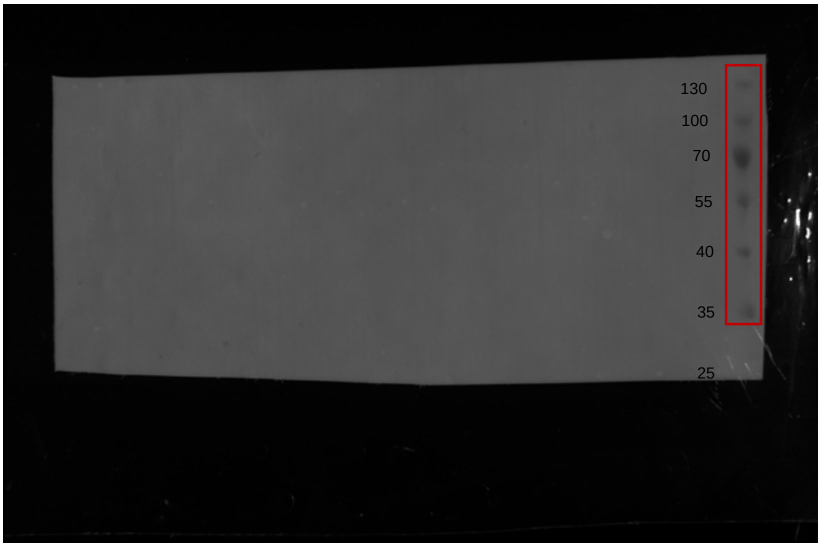

Marker, showing protein weight in kDa. Adjustment used in Fig 13B: increased contrast (+20%) and brightness (+40%), cropped between 130 and 35 kDa, vertical extension.

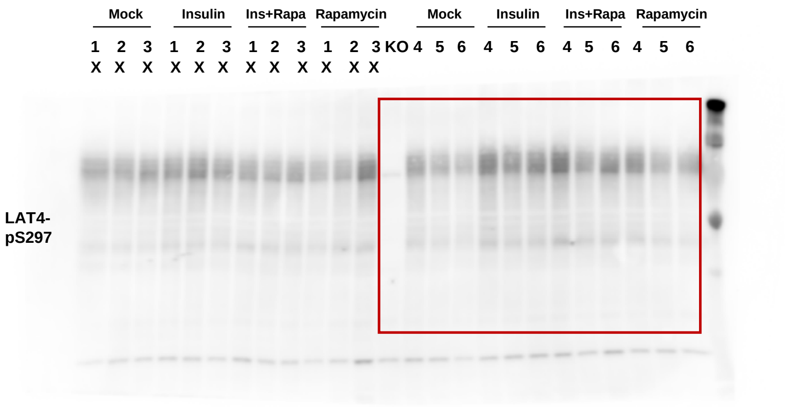

LAT4-pS297 and actin original blot images. Loading sequence shown, same sample numbers indicate same mouse tissue. X marks the lanes not included in the Fig 13B. Adjustments used in Fig 13B: cropped between 130 and 35 kDa, vertical compression and increased contrast (20%) and darkening for LAT4-pS297. Vertical compression for actin.

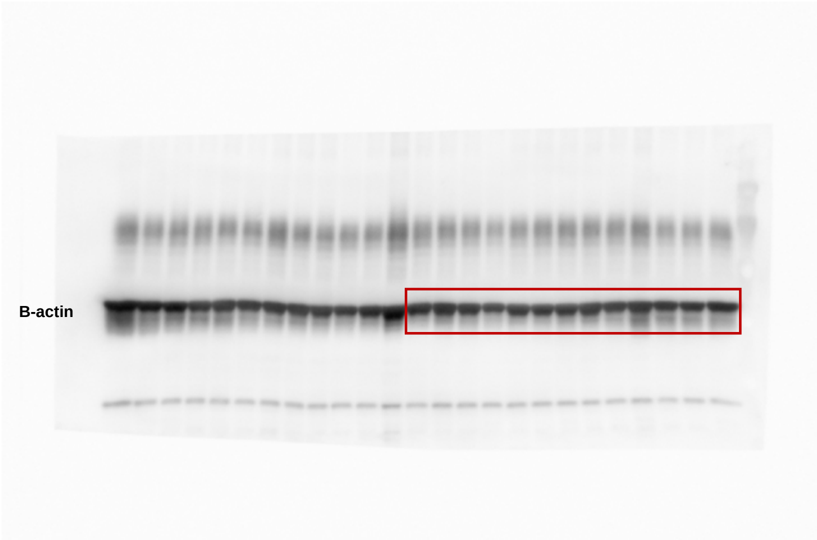

Original blot images from Fig 13C

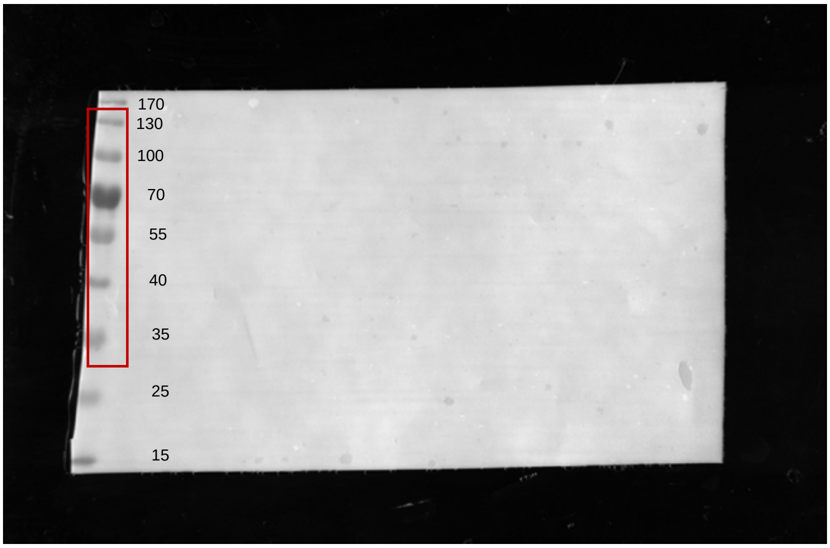

Marker, showing protein weight in kDa. Adjustment used in Fig 13C: increased contrast (+20%), cropped between 130 and 35 kDa, vertical extension.

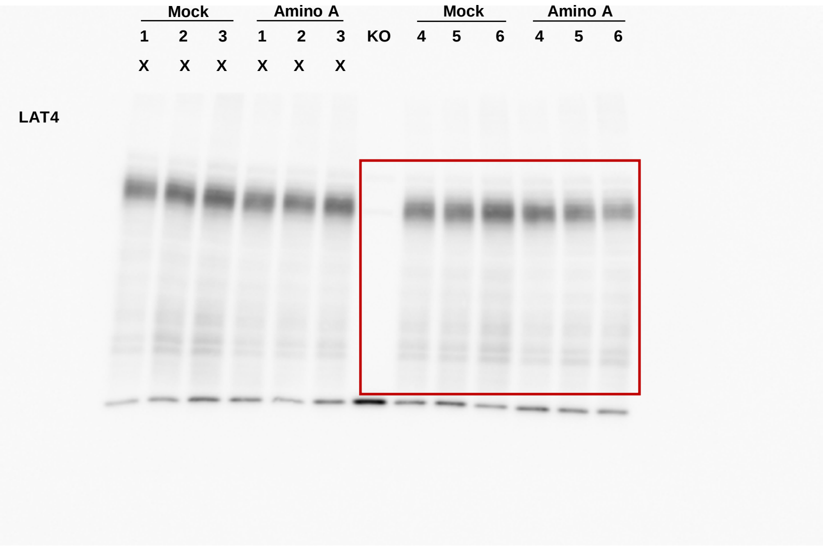

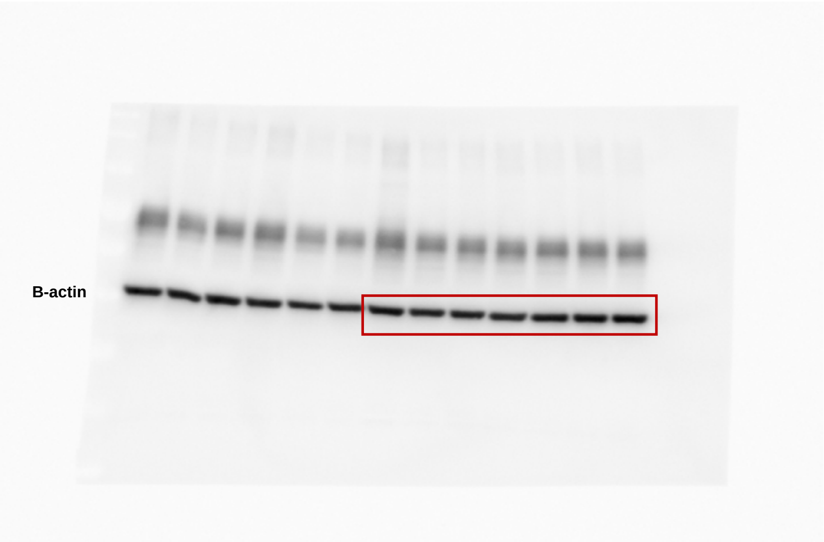

LAT4 and actin original blot images. Loading sequence shown, same sample numbers indicate same mouse tissue. X marks the lanes not included in the Fig 13C. Adjustments used in Fig 13C: cropped between 130 and 35 kDa, vertical compression and increased contrast (20%) for LAT4. Horizontal extension for both.

Original blot images from Fig 13D

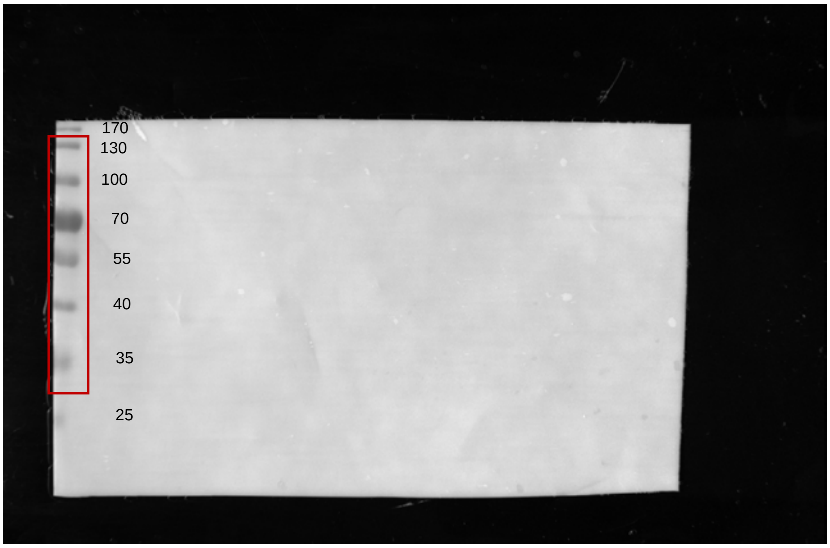

Marker, showing protein weight in kDa. Adjustment used in Fig 13D: increased contrast (+20%), cropped between 130 and 35 kDa, vertical extension.

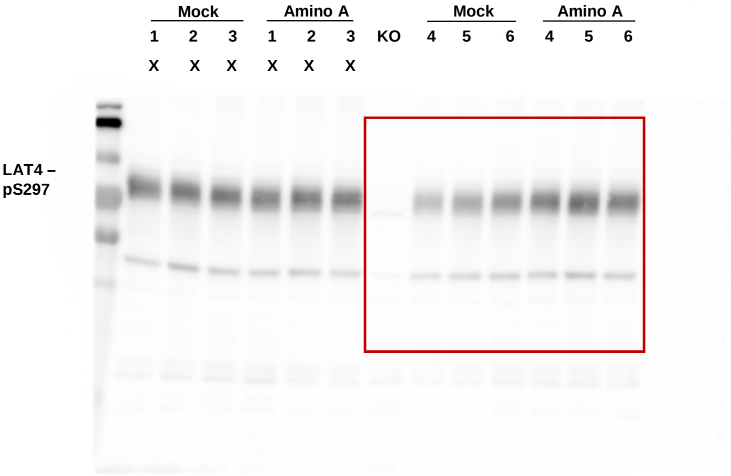

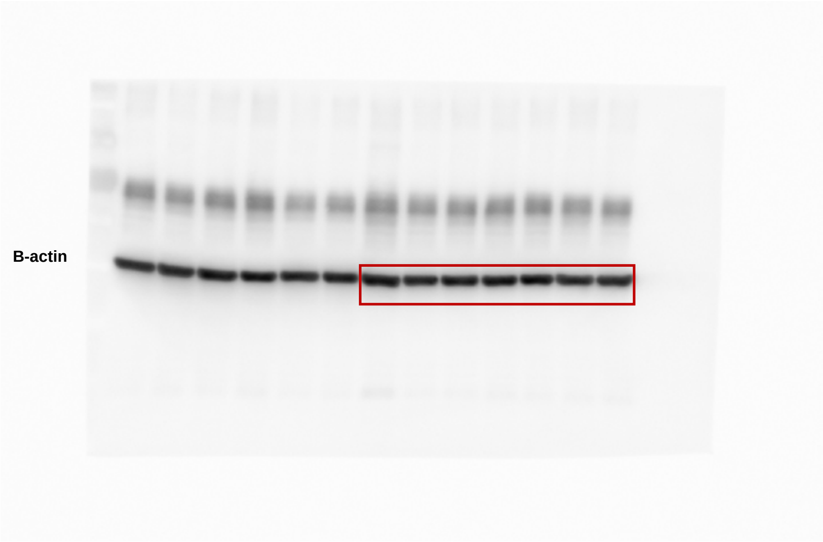

LAT4-pS297 and actin original blot images. Loading sequence shown, same sample numbers indicate same mouse tissue. X marks the lanes not included in the Fig 13D. Adjustments used in Fig 13D: cropped between 130 and 35 kDa, vertical compression and increased contrast (20%) for LAT4-pS297. Horizontal extension for both.
