# Supplementary material for: Phosphorylation of mouse intestinal basolateral amino acid uniporter LAT4 is controlled by food-entrained diurnal rhythm and dietary proteins
Source: PLoS One. 2020 May 29;15(5):e0233863. doi: 10.1371/journal.pone.0233863 (PMC7259769; doi:10.1371/journal.pone.0233863)
Supplement: Attachment — Submitted filename: plos one review.pdf [file pone.0233863.s003.pdf]

Most research on circadian entrainment to feeding has focused on neural mechanisms and the liver, so it is quite interesting to learn about changes in the intestine in the study of Oparija-Rogenmozere and colleagues. They describe the expression of uni-, sim- and anti-porters in response to scheduled feeding and increased/decreased dietary protein content, eventually focusing their study on Lat4, an amino acid transporter that they have studied previously that is regulated by phosphorylation. In the present study they examine the expression and regulation of this transporter in great deal and the study appears technically sound in all ways.

I have several suggests for edits and clarification below:

1. Introduction, line 1, the word “laying” should be deleted
2. Most of the uses of the word “already” are unnecessary and it can be deleted without changing the meaning of the sentences
3. Introduction sentence: “Small intestine also possesses a self-sustaining circadian rhythm for which the most powerful entrainment is food intake (25, 26).” Reference 26 is not correct.
4. Introduction, last paragraph, please consider citing some of the studies that cast some doubt on the necessity of ghrelin peptide as the mechanism of behavioral anticipation of scheduled meals:
    a. Gunapala et al https://doi.org/10.1371/journal.pone.0018377, which demonstrates that ghrelin deletion does not alter food anticipatory activity in mice
    b. And also Daily et al 2012 doi: 10.1210/en.2011-1464, which dissociates gut peptide entrainment from behavioral entrainment
    c. Also state whether that the ghrelin receptor is expressed in the intestine and that you were seeking to test for a direct action of ghrelin on the intestinal cells
5. Proofread by a native speaker--many sentences are missing the article “The”
6. Table I, replace “gender” with “sex”
7. Methods, was one week of inverted LD really enough to shift light entrained rhythms? Was this checked behaviorally?
8. Methods, It is not necessary to denote “JAX™” when describing C57BL/6J mice as the Jax is implied
9. Methods, “45°C”, remove “C”
10. Results, “Thus, the expression of LAT4 protein could be regulated at the level of translation or post-translationally (e.g. protein stability and/or translocation to membrane).”. This later bit about translocation should be removed as it doesn’t appear that the authors specifically isolated membrane fraction (versus other intracellular compartments) with the low speed centrifugation step that was applied. If more effort was taken to isolate membranes than what is described in the Methods section then it should be described there
11. What is the meaning of “Swiss roll”? Please define

12. There are at least two references to unpublished manuscripts. I believe that it is against the policy of PLoS ONE to cite unpublished manuscripts. At least that was the case the last time I published in PLoS ONE in 2018.
13. Link to phospho web domains needed only in methods section
14. For the “box and whisker” plots, it is only necessary to describe their meaning once instead of repeatedly in each figure legend.
15. Please attempt to fit a sine wave to the data that you believe to be showing a circadian rhythm and describe the goodness of fit to a sine wave. I appreciate that the data points are shown but it would be nice to see if these are rhythmic or not as many of them just appear noisy.
