# Supplementary material for: Phosphorylation of mouse intestinal basolateral amino acid uniporter LAT4 is controlled by food-entrained diurnal rhythm and dietary proteins
Source: PLoS One. 2020 May 29;15(5):e0233863. doi: 10.1371/journal.pone.0233863 (PMC7259769; doi:10.1371/journal.pone.0233863)
Supplement: Attachment — Submitted filename: Response to Reviewers.docx [file pone.0233863.s004.docx]

**PONE-D-20-02951**
Phosphorylation of mouse intestinal basolateral amino acid uniporter LAT4 is controlled by food-entrained diurnal rhythm and dietary proteins
(slightly modified title)

*Lalita Oparija-Rogenmozere et al.*,

**Replies to the points raised by the reviewers**

----------------------------------------------------------------------------------
**Reviewer #1:** Oparija-Rogenmozere and colleagues investigated the expression and phosphorylation of essential amino acid transporter Lat4 (Slc43a2) in three regions of the intestine of mice fed from early to late night. The results indicate differential expression and phosphorylated states of Lat4 according to intestine regions (i.e. duodenum, jejunum and ileum) and amount of proteins in the diet (8, 18 or 40%).
As detailed below, the manuscript raises several methodological and interpretative issues that need to be addressed.

***Reply:*** *Thank you for your careful review containing many useful and constructive comments. The authors feel that the corresponding changes made to the manuscript have clearly improved its quality.*

1. Food-entrained versus ad lib-fed mice
It is not fully clear why all mice had food access limited to 8 h, starting at dark onset. Here food access is limited to the usual feeding period which might have enhanced daily rhythmicity. Why were mice fed ad libitum not studied as control group? Otherwise, what is the advantage or the purpose to study food-restricted mice fed only at night? Please specify.

***Reply:*** *We have used a time restricted feeding (TRF) protocol to enhance the diurnal rhythmicity. It is indeed known from the literature that for the small intestine and other peripheral organs, food intake is a more powerful synchronizer of the daily rhythm than the SCN and that timed food restriction is essential for the appearance of food-anticipatory activity (See review by Pendergast & Yamazaki. The mysterious food-entrainable oscillator: Insights from mutant and engineered mouse models. J Biol Rhythms. 2018; 33: 458-474). Also, as shown in the paper by Greenwell et al. (Rhythmic food intake drives rhythmic gene expression more potently than the hepatic circadian clock in mice. Cell Rep. 2019; 27:649-657), mice that are fed only during the night show ~30% more rhythmically expressed genes in liver than mice fed ad libitum (unfortunately no analogue study has been done for the intestine). Thus, we expected that TRF may act as zeitgeber for the intestinal clock, leading to more coordinated responses as ad libitum feeding.*

## *The aim of the present study was to further characterize the LAT4 phosphorylation changes, in particular in response to food anticipation, food intake and resting phase. Animals fed ad libitum included in our previous study on LAT4 phosphorylation (doi: 10.1113/JP276714) had shown a lack of food-anticipatory response and were not further characterized. We show here for the reviewer a full version of the previously published blot, where the low level of S297 phosphorylation at ZT12 (time of food anticipation) in 2 ad lib fed animals can be seen. Therefore, in this study only TRF mice were used, with the ZT0 (lights on) timepoint used as control timepoint for the diurnal study and the 18% protein diet used as control for the dietary study. This has been clarified in the Materials and Methods section in paragraph “Animal housing and food entrainment for ghrelin, diurnal and dietary regulation experiments”.*

*Thus, in view of the previously published data and the aims of the present study, we consider that adding an ad libitum group would not strengthen our message. For technical reasons, this could not be added to the study, since the laboratory of the senior author F. Verrey has been recently closed due to his mandatory retirement and the current labs of the first and the second author are anyway closed due to the Covid-19 pandemic.*

***Fig. 1 for reviewers.*** *Full version of the previously published Western blot showing two lanes with ad libitum fed mice*

Fig_Rev 1. LAT4 phosphorylation on S297 at ZT12 (food expectation) in Ad libitum fed (AL) and time-restricted fed (TRF) mice. LAT4 knockout (KO) mice demonstrate the specificity of LAT4-pS297-specific antibody.

2. Circadian control
Reading the present manuscript raises comments on the concept of circadian clocks. Circadian rhythms refer to endogenous biological rhythms that are controlled by self-sustained clocks. Such endogenopus rhythms can only be assessed in constant environmental conditions (including constant light or dark, and constant ambient temperature). When a light-dark cycle is present, as it is the case in the present study, the parameters studied over 24 h should be called « daily » or « diurnal » rhythms. As a consequence, the word « circadian » such as used in the title is inappropriate for the present experiment and should be omitted throughout the manuscript.

***Reply:*** *Thank you for the correction, we agree that daily or diurnal is the correct term to use for the rhythmic changes we observed. “Circadian” was replaced with “daily” or diurnal throughout the manuscript.*


3. Clock-controlled versus feeding-induced regulation
This study reports daily variations of several parameters such as protein levels of LAT4 in the duodenum and LAT4-pS297 in jejunum. When higher values are found at night, the present protocol does not allow discriminating between putative clock-controlled or feeding-induced regulation.
To test for feeding-induced control, another group of mice should be fed during daytime only (with the assumption that there will be a 12-h shift in the daily variations). The same remark holds for food-anticipatory mechanisms.
To test for clock-controlled regulation, another group of mice should be fasted (with the assumption that the daily variations will persist if they are regulated by endogenous clocks).
If ever the authors cannot add these new groups of mice, they should be much more cautious in their discussion on the interpretation of their findings, so as not to mislead the reader.

***Reply:*** *We agree with the suggestion of the reviewer to be more cautious regarding the interpretation of our findings and we have correspondingly made changes in the Discussion that make it clearer that LAT4 phosphorylation responds to the food-entrained diurnal rhythm. This interpretation / statement includes the possible role of both local and central clock effects – in any case, TRF acts as a zeitgeber for synchronisation.*

*It this context it is useful to recall that the mammalian clock operates on multiple hierarchy levels and that thus a simple distinction between clock-induced and feeding-induced control is experimentally not easily feasible (See Patke et al. Molecular mechanisms and physiological importance of circadian rhythms. Nat Rev Mol Biol. 2020; 21:67-84 and Patcha & Sumova. Circadian regulation of epithelial functions in the intestine. Acta Physiol. 2013; 208: 11-24). We indeed agree that the protocol we used does not distinguish between central and peripheral clock effects and adapted, as mentioned above, the Discussion of our results. As mentioned above we are for technical reasons not in the position of adding new experimental groups. Furthermore, we would fear that the suggested experimental protocols such as daytime feeding or fasting of the animals would, by introducing deleterious factors, potentially confuse the results (see for instance: Codoner-Franch & Gombert. Circadian rhythms in the pathogenesis of gastrointestinal diseases. World J Gastroeneterol. 2018; 24(38):4297-4303 and Voigt et al. Circadian rhythms: a regulator of gastrointestinal health and dysfunction.* Expert *Rev Gastroenterol Hepatol. 2018; 13(5): 411-424). An experimental approach that eventually could demonstrate the role of food-entrained peripheral clocks versus the central clock would be the use of mice with lesioned SCN (central pacemaker), but this would of course be beyond the possible scope of this study.*

4. Image analysis and quantification
In figures 9 and 11, the authors consider that « low protein diet leads to increased intracellular localization of LAT4 in jejunum » and this diet « has no strong impact on LAT4 subcellular localization in ileum ». It seems that these respective statements are based on visual inspection only. Image analysis and quantification are required for both parameters.

***Reply:*** *Indeed, the original statements were based on visual inspection and we appreciate the suggestion to quantify this observation. We have now analysed the images and assessed the Pearson’s colocalization coefficients as described in the Methods section under 'Image Analysis'. The results confirm our previous visual observation and have been added to Fig. 9 and 11.*

5. Circadian clock in the intestine
It is surprising for a study supposed to investigate clock-related variations in the intestine that the authors did not cite previous investigations of circadian oscillations in that organ. Actually, the 2 proposed citations (one original work and one review) do not deal at all with the intestinal clock (i.e. Damiola et al. 2000 and Welsh et al. 2010, see refs 25 and 26, respectively).

Alternative suggestions:
Hepatic, duodenal, and colonic circadian clocks differ in their persistence under conditions of constant light and in their entrainment by restricted feeding. Polidarová L, Sládek M, Soták M, Pácha J, Sumová A. Chronobiol Int. 2011 Apr;28(3):204-15. doi: 10.3109/07420528.2010.548615.
PMID:21452916

Clock gene expression in the murine gastrointestinal tract: endogenous rhythmicity and effects of a feeding regimen. Hoogerwerf WA, Hellmich HL, Cornélissen G, Halberg F, Shahinian VB, Bostwick J, Savidge TC, Cassone VM. Gastroenterology. 2007 Oct;133(4):1250-60. Epub 2007 Jul 12. PMID: 17919497

***Reply:*** *We included references (References #25-27 in the Manuscript) that describe circadian clocks in the small intestine, including the first of the suggested ones (the second (Hoogerwerf et al.) focuses on stomach and colon). We also updated the description of the intestinal clock in the Introduction.*


6. Miscellaneous
The main text indicates that mice were fed from ZT12 to ZT20, while Figure 1 suggests that mice had food access from ZT12 to ZT18. Please correct where needed.

***Reply:*** *Thank you! This was a mistake in Figure 1 which we have corrected.*

« Rest » or « resting » period, rather than « passive » period.

***Reply:*** *OK, corrected.*

----------------------------------------------------------------------------------

**Reviewer #2:** Most research on circadian entrainment to feeding has focused on neural mechanisms and the liver, so it is quite interesting to learn about changes in the intestine in the study of Oparija-Rogenmozere and colleagues. They describe the expression of uni-, sim- and anti-porters in response to scheduled feeding and increased/decreased dietary protein content, eventually focusing their study on Lat4, an amino acid transporter that they have studied previously that is regulated by phosphorylation. In the present study they examine the expression and regulation of this transporter in great deal and the study appears technically sound in all ways.

***Reply:*** *Thank you for your positive statements and your constructive suggestions and corrections which clearly led to an improvement of the manuscript and its readability.*

I have several suggests for edits and clarification below:

1. Introduction, line 1, the word “laying” should be deleted

***Reply:*** *Word “laying” was deleted.*

2. Most of the uses of the word “already” are unnecessary and it can be deleted without changing the meaning of the sentences

***Reply:*** *In the majority of the cases the word "already" has now been deleted.*

3. Introduction sentence: “Small intestine also possesses a self-sustaining circadian rhythm for which the most powerful entrainment is food intake (25, 26).” Reference 26 is not correct.

***Reply:*** *Thank you! This section of the Introduction has been rewritten, using more appropriate references.*

4. Introduction, last paragraph, please consider citing some of the studies that cast some doubt on the necessity of ghrelin peptide as the mechanism of behavioral anticipation of scheduled meals:
Gunapala et al <https://doi.org/10.1371/journal.pone.0018377,> which demonstrates that ghrelin deletion does not alter food anticipatory activity in mice
And also Daily et al 2012 doi: 10.1210/en.2011-1464, which dissociates gut peptide entrainment from behavioral entrainment
Also state whether that the ghrelin receptor is expressed in the intestine and that you were seeking to test for a direct action of ghrelin on the intestinal cells

***Reply:*** *We added more information on ghrelin and food anticipation in the introduction. However, as you mention, our study focuses rather on the impact of food intake on gene expression/post-translational modifications of amino acid transporters in intestinal cells. The fact that the study by Daily et al. detected an increase in plasma ghrelin in food-entrained rats at expected meal intake time actually supports our decision to investigate whether ghrelin as a regulated hormone/factor could induce the observed changes of amino acid transporter LAT4 phosphorylation.*

*We improved the description of our ghrelin experiments to indicate more clearly that we were looking for a direct action of ghrelin on the intestinal mucosa by in vivo injections. We now also mention in the Introduction what is known about the expression of ghrelin receptors in the small intestine.*

5. Proofread by a native speaker--many sentences are missing the article “The”

***Reply:*** *Thank you, the use of articles was carefully reviewed*

6. Table I, replace “gender” with “sex”

***Reply:*** *Thanks, replaced!*

7. Methods, was one week of inverted LD really enough to shift light entrained rhythms? Was this checked behaviorally?

***Reply:*** *We have adjusted the text in the revised manuscript to explain better how the shift to inverted LD was done. Actually 1-week adjustment to the inverted LD meant that mice were first kept for 1 week with inverted LD cycle while still fed ad libitum to avoid the stress of changing both LD and feeding rhythms at the same time. Afterwards we stopped ad libitum feeding and introduced time-restricted feeding for 14 days, all still in the inverted LD. Thus, all mice spent at least 3 weeks in inverted LD rhythm. Studies on clock genes Per1, Per2 and Cry1 in SCN indicate that adjustment to phase shift of LD cycle (10 hrs, so almost reversed) happens within 5-7 days (See Nagano et al. An abrupt shift in the day/night cycle causes desynchrony in the mammalian circadian center. J Neurosci. 2003; 23(14): 6141-6151).*

*Regarding the circadian clocks in the small intestine, it has been shown in many studies that food-entrainment is a much more powerful zeitgeber than light (For review see Pacha & Sumova. Circadian regulation of epithelial functions in the intestine. Acta Physiol. 2013; 208: 11-24). Therefore, we were confident that two weeks of food entrainment would be sufficient to trigger possible diurnal changes of the amino acid transporters of interest.*

8. Methods, It is not necessary to denote “JAX™” when describing C57BL/6J mice as the Jax is implied

***Reply:*** *JAX was removed from the strain name in the text.*

9. Methods, “45^o^C”, remove “C”

***Reply:*** *Removed.*

10. Results, “Thus, the expression of LAT4 protein could be regulated at the level of translation or post-translationally (e.g. protein stability and/or translocation to membrane).”. This later bit about translocation should be removed as it doesn’t appear that the authors specifically isolated membrane fraction (versus other intracellular compartments) with the low speed centrifugation step that was applied. If more effort was taken to isolate membranes than what is described in the Methods section then it should be described there

***Reply:*** *Regarding the food-entrained mice, the isolated intestinal villi were processed for total membrane isolation. We apologize that the description was not included in the Methods section. The original manuscript included the postprocessing description of only ex-vivo treated villi.*

*We have adjusted the Methods section accordingly (Under Preparation of intestinal fractions) and the Result section. We have also removed the statement 'or translocation to the membrane' (not relevant in this context).*

11. What is the meaning of “Swiss roll”? Please define

***Reply:*** *We used the term 'Swiss roll' because this term was coined in the first description in 1981 of this simple technique used for histological studies of rodent intestine (See Moolenbeek & Ruitenberg. The “Swiss roll”: a simple technique for histological studies of the rodent intestine. Lab Animals. 1981; 15). Shortly, intestine is rolled after the collection and fixed in this way before OCT embedding. It allows to embed long, uninterrupted segments of the small intestine to examine changes along the longitudinal axis. This method is superior to embedding just short pieces of intestine or intestinal rings, because larger areas and higher numbers of villi can be analysed. See the schematic drawing below. We have adjusted the Swiss roll description in Methods section (Immunofluorescence subsection) to stress its advantages.*

**A**

**B**

**C**

*A: Intestine embedded as Swiss roll. B: Intestine embedded as intestinal rings – lacks the continuity and covers less area. C: intestine embedded as multiple short segments – also the same caveats as for B.*

12. There are at least two references to unpublished manuscripts. I believe that it is against the policy of PLoS ONE to cite unpublished manuscripts. At least that was the case the last time I published in PLoS ONE in 2018.

***Reply:*** *We actually do not know whether PLoS ONE accepts the mention of a 'manuscript in preparation' or 'unpublished observations' that does not appear in the reference list (that of course would not be acceptable according to the Submission guidelines). Actually, the three 'references' were to the same manuscript that is currently under preparation and shares multiple authors with this manuscript. This yet unpublished manuscript describes the specific animal knockout strains that were used as controls in this study (e.g. why these tissue-specific Cre promoters were used, the efficiency of LAT4 knockout, etc..). We had felt that it may be useful to announce that this underlying data will be published. However, since we describe the origin and source of the animal strains in the current Manuscript in enough detail, we decided to remove these references from two places in Materials and methods and to replace it with 'A. Rajendran, unpublished observation' in the Discussion.*

13. Link to phospho web domains needed only in methods section

***Reply:*** *Links are now given only in the Methods section (Ex vivo treatments subsection) and were removed from the other parts of the manuscript.*

14. For the “box and whisker” plots, it is only necessary to describe their meaning once instead of repeatedly in each figure legend.

***Reply:*** *The description of the meaning of this plot was left in the first figure where it appears (Fig. 5) and omitted from other legends.*

15. Please attempt to fit a sine wave to the data that you believe to be showing a circadian rhythm and describe the goodness of fit to a sine wave. I appreciate that the data points are shown but it would be nice to see if these are rhythmic or not as many of them just appear noisy.

***Reply:*** *this is a very good suggestions: We used the online program Cosinor.Online (see new Methods) to test in our datasets the presence and the significance of diurnal sinusoidal oscillations. The fit of the cosine wave was assessed by performing a Zero-amplitude test. We have added the cosine wave fits to our graphs and adjusted the manuscript accordingly.*

--------------------------------------------------------------------------------------------------------------------------
